# Detection of *Toxoplasma gondii* by PCR and Mouse Bioassay in Rodents of Ahvaz District, Southwestern Iran

**DOI:** 10.1155/2014/383859

**Published:** 2014-01-29

**Authors:** J. Saki, S. Khademvatan

**Affiliations:** ^1^Department of Medical Parasitology, Ahvaz Jundishapur University of Medical Sciences, Ahvaz 613715794, Iran; ^2^Health Research Institute, Infectious and Tropical Diseases Research Center, Ahvaz Jundishapur University of Medical Science, Ahvaz, Iran; ^3^Research Institute for Infectious Diseases of Digestive System, Ahvaz Jundishapur University of Medical Sciences, Ahvaz 613715794, Iran

## Abstract

*Toxoplasma gondii* is obligate coccidian zoonotic parasite. Felidae family is definitive and wide ranges of warm-blooded vertebrates are intermediate hosts for the parasite. Rodents are measured as an important source of *T. gondii* infection for the definitive host. Thus, this study aimed to investigate *Toxoplasm* infection in rodents of Ahvaz district, southwest of Iran. A total of 100 rodents (73 *Rattus norvegicus*, 21 *Rattus rattus*, and 6 *Mus musculus*) were collected and studied by GRA6PCR and mouse bioassay. The finding indicated that 6 out of 100 (6%) and 2 out of 100 (2%) samples were positive by PCR and mouse bioassay, respectively. The results show notable chronic infection in the rodent and potential transmission of the infection among animal and men in the region. Accordingly, this study recommended investigating of the *T. gondii* infection in definitive and other intermediate hosts in other points of Khuzestan province, Southwest, Iran.

## 1. Introduction

*Toxoplasma gondii* is an intracellular and obligate coccidian zoonotic parasite. The definitive hosts of the parasite are members of the Felidae family (mainly domestic cats) and the intermediate hosts are warm-blooded vertebrates including humans, rodents, birds, livestock, and marine mammals [1].

Felids with excreting of the environmentally resistant oocysts are the most important hosts in the life cycle of *T. gondii*. Cats become infected with *T. gondii* by eating infected tissues from intermediate hosts. While foraging on the ground, rodents can get infected by *Toxoplasma gondii*. Toxoplasmosis is one of the opportunistic infections which in people with immunocompetent is largely asymptomatic, but in immunocompromised individuals, the parasite can become widely disseminated, causing severe toxoplasmosis [2–4]. In Iran, toxoplasmosis continues to be a public health problem, and a seroprevalence of 41.4% has been reported in central part of the country [5].

Assessment of *Toxoplasma* infection in the rodents as a main prey for cats with a key role in the ecological food chain and also in the transmission of parasites to other animals is very important. Studies in Iran revealed existence of different species of rodent including the brown rat (*Rattus. norvegicus*), the black rat (*R*. *rattus*), the Himalayan rat (*R. pectoris*), and house mice, *Mus musculus* [6, 7]. In Ahvaz, Southwest Iran, mainly due to unsupervised housing constructions and lack of health sewage system, large numbers of wild and domestic rodents are found on residential streets. These populations are potentially an important host reservoir for the transmission of zoonotic parasites such as *T*. *gondii* [8]. Although several studies were conducted on *Toxoplasma* infection in rodents in Iran [6, 7, 9], there is no report of molecular and change for mouse bioassay studies of *Toxoplasma* isolates from southwest of Iran.

The polymerase chain reaction (PCR) is very sensitive, highly specific, and rapid molecular technique to detect the parasite animal and human tissues [10]. Mouse bioassay is the principal method used to detected cysts in tissues [11]. The objective of this study was to determine *Toxoplasma* infection in domestic and wild rodents in Ahvaz, southwest of Iran by bioassay in mice, polymerase chain reaction (PCR), and sequencing.

## 2. Materials and Methods

### 2.1. Geographical Information on Study Area

Ahvaz is the capital of Khuzestan province, southwest of Iran. At the 2006 census, its population was 1,425,891, in 212,097 families. The city is built on the banks of the Karun River and has an average elevation of 20 meters above sea level. Ahvaz has a desert climate with long, extremely hot summers and mild, short winters. Maximum temperature in summer routinely exceeds 50 degrees while in winters the minimum temperature could fall around −5 degrees Celsius. The average annual rainfall is around 230 mm. In Ahvaz there is high density of rodents and rat-man proximity is considerable [7].

### 2.2. Rodent Collection

In the year 2011, trappings were performed at different parts of the city using live traps. In each locality, traps were baited with favorite foods of rodents at different seasons. On consecutive days, traps were collected and transported to medicine school, Parasitology department, where rodents were bled. Species identification and sex and age determination were done as described by Roberts [12].

### 2.3. Restraining of the Rodents and Tissue Collection

The trapped rodents were anaesthetized by putting the live traps in a thick transparent polythene bag and then a cotton swab soaked in ether as described by Singla et al. [13].

A total of 100 rodents (73 *Rattus norvegicus*, 21 *Rattus rattus*, and 6 *Mus musculus*) were bled and following microscopic examination for the presence of tissue cysts, all the tissues were artificially digested (0.5% pepsin, 1% HCl) [14–16].

### 2.4. Detection of *T. gondii* in Tissue by the Polymerase Chain Reaction and Sequencing

DNA was extracted from digested-tissue samples of brain, skeletal muscle, and liver using Bioneer Co., Seoul, South Korea, according to the manufacturer's instructions. Purified DNA concentration was determined by UV absorption [17].

For positive control, confirmed genomic DNA (Genbank accession number AB733004) was used in PCR amplification.

Detection of *T. gondii* DNA was carried out using a single PCR assay targeting the GRA6 region. For *T. gondii*-specific amplification, two forward primer, 5′- GTAGCGTGCTTGTTGGCGAC-3′, and reverse primer, 5′-ACAAGACATAGAGTGCCCC-3′, primers were used as described by Fazaeli et al. [18].

The PCR reaction was performed in a 25 *μ*L reaction mixture containing 10 *p*mol of each primer and 10 *μ*L of extracted DNA, 75 mM Tris-HCl (PH 8.5), 20 mM (NH_4_)_2_SO_4_, 1.5 mM MgCl_2_, 0.1% Tween 20, 0.2 mM dNTPs, 0.025 unite/*μ*L Amplicon Taq DNA polymerase, inert red dye, and a stabilizer. The PCR conditions were 5 min at 95°C followed by 35 cycles of 30 s at 94°C, 1 min at 60°C, 2 min at 72°C, and a final elongation of 72°C for 7 min [18].

The PCR products were separated on a 1.5% agarose gel with 1x TAE buffer and visualized by staining with ethidium bromide.

The PCR positive samples were purified using an Accuprep Gel Purification kit (Bioneer, Deajeon, Korea) then sequenced (MWG-Biotech, Ebersberg, Germany) by the primers employed in the PCR. Sequence alignments were constructed using the program CLUSTAL W version 1.83 (http://www.ddbj.nig.ac.jp/search/clustalwe.html).

### 2.5. Bioassay in Mice

White laboratory mice weighing 20–25 g with 6-week-old specific-pathogen free were used in the bioassay. Tissue samples of liver, muscle, and brain from trapped rodent were submitted to artificial digestion and 1 mL of each tissue homogenate was intraperitoneally inoculated into 2 mice per each tissue sample. Mice were daily inspected for signs that might indicate acute toxoplasmosis. Mice were killed 50 days after infection and the brain of all inoculated mice was studied by direct microscopy for presence of *Toxoplasma gondii* cysts.

## 3. Results and Discussion

Brain, muscle, and liver tissues of 100 wild and domestic rodents were analyzed by GRA6PCR method. PCR showed 6/100 (6%) positive samples (4 from *Rattus norvegicus* and 2 from *Rattus rattus*), being all from brain tissues. No positive result was detected from *Mus musculus* species. Of the 4 infected *Rattus norvegicus* isolates, 3 were collected from urban points and one was from rural region, whereas both of the infected *Rattus rattus* ones were collected from urban sit. This finding indicated that *Rattus norvegicus* and *Rattus rattus* could be a significant source of *T. gondii* infection for stray cats in the urban region.

In addition, No PCR-positive value was found for muscle and liver tissues. Tissue cysts of *T. gondii* were identified in 2 brain tissues of inoculated mice studied by direct microscopy examination (Table 1).

**Figure 1:**
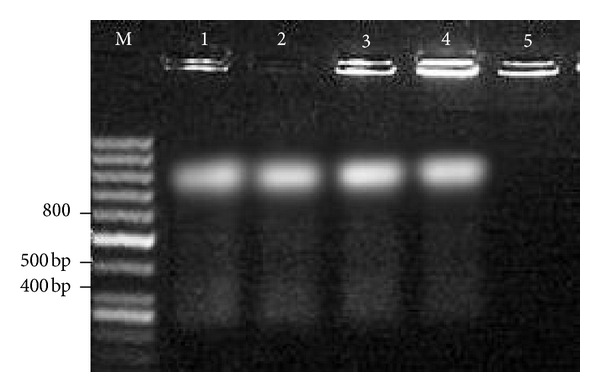
PCR amplification products of *Toxoplasma gondii* GRA6 in Rodent tissue samples. Lanes: M: molecular weight marker; 1: positive control (*Toxoplasma* tachyzoites); 2, 3, 4: positive samples; 5: negative control (H_2_O dest instead of DNA).

The size of the PCR products was about 800 bp [18] (Figure 1). The finding of GRA6 PCR technique indicated highest rate of *T. gondii* infection in *Rattus norvegicus* with 4/73 (5.4%).

**Figure 2:**
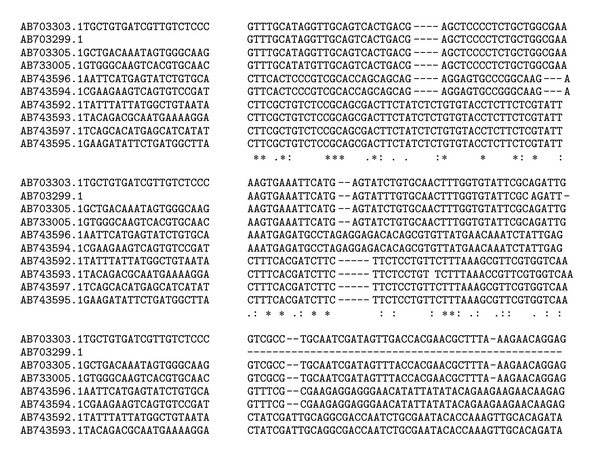
Multiple alignments of nucleotide sequences of GRA6 gene from 6 isolates and 4 nucleotide sequences of *T. gondii* (GenBank accession nos. AB703303, AB703299, AB703305, and AB733005). Asterisks indicates identical nucleotides.

The amplified GRA6 genes of the 6 isolates were sequenced. The results were submitted to DDBJ/Genbank at accession nos: AB743592–AB743597. The nucleotide sequences of GRA6 gene from isolates were aligned with nucleotide sequences of *T. gondii* (GenBank accession nos. AB703303, AB703299, AB703305, and AB733005) (Figure 2). Figure 2 showed different nucleotide substitutions. These sequences were previously detected in domestic and wild birds in the region [19]. The obtained GRA6 sequences indicated atypical type of *T. gondii* in the region. This result suggested that there are probably different *T. gondii* genotypes circulating in this region.

Two positive brain samples of inoculated lab mice, detected by direct microscopic observation, were also studied by PCR technique. The obtained sequences were recorded as AB743592.1 and AB743593.1 accession numbers. Comparisons of these sequences with sequences that have been previously obtained from brain samples of hunted rodents (inoculated in the lab mice) showed 100% homology.

In this study, we used a PCR and sequencing at GRA6 geneand bioassay in mice for detection of *T. gondii* infection in rodent populations in Ahvaz, southwest Iran. Finding of this investigation indicated 6% of *T. gondii* infection in Ahvaz rodent which is in accordance with previous reports in Tehran and Zanjan, Iran, which showed 5.5% (8/145) of *T. gondii* infection in mouse brain tissues by PCR-RFLP. Seroprevalence study of *Toxoplasma gondii* among wild Rats (*Rattus rattus*) in Ahvaz district, Southwestern Iran, in 2011 showed thirty-one of the 127 serum samples (24.41%) had antibodies against *T. gondii* [9]. It could be due to the kind of test which seroprevalence results indicating exposure to *T. gondii* and not still associated with forming cysts in tissues.

In the present study, in bioassay method, *T. gondii* was isolated from the brain of two lab mice inoculated brain suspensions of trapped mice. This result is corresponding with Sreekumar et al. [20] in bioassay of chicken tissues in mice who reported that* T. gondii* could not be isolated from any of the positive samples by bioassay in mice, whereas they could isolate *T. gondii* from the feces of five cats fed tissues pooled from 89 chickens with titers of 1 : 5 or less. The lower prevalence rate in this assay could be due to various reasons. It is possible that the samples that were analyzed herein originated from younger mice. It is likely that storage of samples under unrefrigerated conditions during preparation affected the viability of *T. gondii*. PCR is a sensitive and specific for detecting *T. gondii* even when the tissues available for testing are in state of decomposition; bioassays, in contrast, can only detect viable parasites [10, 21, 22]. The present results are similar to Yai et al. [10] who detect lower levels of identification via bioassays (four out of eight pigs) than with nested-PCR (seven out of eight pigs). However, many studies worldwide reported higher sensitivity for murine bioassays than PCR with respect to the isolation of *T. gondii* [23–25]. The present results showed that the brain was the most frequently infected compared with the other tissues. These results concur with those of Esteban-Redondo and Innes [26].

In this study, the most abundant species was *Rattus norvegicus* (73/100) and highest prevalence of *T. gondii* infection (5.4%, 4/73) was detected in this species. This finding is in agreement with previous study in Ahvaz that indicated* Rattus norvegicus* in 80% of the trapped rodent [7]. The highest rate of *T. gondii* infection detected in* Rattus norvegicus* may be due to the fact that repeat congenital transmission happens in this species [27]. Nonetheless, recent studies have used PCR in The Netherlands and the United Kingdom demonstrating a very high prevalence in *Mus musculus* (51.9%, 120/231) and lower in *R. norvegicus* (10.3%, 4/39) [28, 29].

## 4. Conclusions

This is the first molecular and bioassay survey of *T*. *gondii* infection among wild and domestic rodent in Ahvaz district, southwestern Iran, and the results indicate notable chronic infection in the rodent and potential transmission of the infection among animal and men. We suggest the study of the *T. gondii* infection in definitive and other intermediate hosts in other points of Khuzestan province, Southwest, Iran.

## Figures and Tables

**Table 1:** The results of *Toxoplasma gondii* detection in rodent of Ahvaz, southwest Iran, using PCR, mouse bioassay, and direct microscopy examination methods.

Species	*N*	PCR	Mouse bioassay	Direct microscopy examination	Total positive samples
*Rattus rattus *	21	2 (9.5%)	0	1	2
*Rattus norvegicus *	73	4 (5.4%)	2 (2.7%)	0	4
*Mus musculus *	6	0	0	0	0

Total	100	6%	2%	1%	6
